# Association between Exposure to Electromagnetic Fields from High Voltage Transmission Lines and Neurobehavioral Function in Children

**DOI:** 10.1371/journal.pone.0067284

**Published:** 2013-07-03

**Authors:** Jiongli Huang, Tiantong Tang, Guocheng Hu, Jing Zheng, Yuyu Wang, Qiang Wang, Jing Su, Yunfeng Zou, Xiaowu Peng

**Affiliations:** 1 Center for Environmental Health Research, South China Institute of Environmental Sciences, Ministry of Environmental Protection, Guangzhou, Guangdong, China; 2 School of Public Health, Guangxi Medical University, Nanning, Guangxi, China; 3 School of Public Health and Tropical Medicine, Southern Medical University, Guangzhou, Guangdong, China; 4 Environmental Health and Related Product Safety, Chinese Center for Disease Control and Prevention, Bejing, China; Aga Khan University, Pakistan

## Abstract

**Background:**

Evidence for a possible causal relationship between exposure to electromagnetic fields (EMF) emitted by high voltage transmission (HVT) lines and neurobehavioral dysfunction in children is insufficient. The present study aims to investigate the association between EMF exposure from HVT lines and neurobehavioral function in children.

**Methods:**

Two primary schools were chosen based on monitoring data of ambient electromagnetic radiation. A cross-sectional study with 437 children (9 to 13 years old) was conducted. Exposure to EMF from HVT lines was monitored at each school. Information was collected on possible confounders and relevant exposure predictors using standardized questionnaires. Neurobehavioral function in children was evaluated using established computerized neurobehavioral tests. Data was analyzed using multivariable regression models adjusted for relevant confounders.

**Results:**

After controlling for potential confounding factors, multivariable regression revealed that children attending a school near 500 kV HVT lines had poorer performance on the computerized neurobehavioral tests for Visual Retention and Pursuit Aiming compared to children attending a school that was not in close proximity to HVT lines.

**Conclusions:**

The results suggest long-term low-level exposure to EMF from HVT lines might have a negative impact on neurobehavioral function in children. However, because of differences in results only for two of four tests achieved statistical significance and potential limitations, more studies are needed to explore the effects of exposure to extremely low frequency EMF on neurobehavioral function and development in children.

## Introduction

Whenever electricity is generated, transmitted or consumed, electromagnetic fields (EMF) are created. The abundant use of high voltage transmission (HVT) lines has resulted in much concern being raised about the impact of EMF exposure from HVT lines on human health, especially in children [1]. As a result, construction of new HVT lines has met considerable opposition in some countries [2]. Over the last thirty years, the possible relationship between EMF exposure from HVT lines and childhood cancer has been a constant topic of interest since first reported by Wertheimer and Leeper in 1979 [3]. Based on studies in children with leukemia, the International Agency for Research on Cancer (IARC) has classified extremely low frequency electromagnetic fields (ELF-EMF) as a possible carcinogen [4].

Studies investigating potential causal associations between exposure to ELF-EMF and adverse health outcomes have mostly focused on childhood cancers [5]–[7] or nervous system diseases, such as amyotrophic lateral sclerosis [8]–[11], brain tumors [12], [13] and Alzheimer’s disease [14], from occupational exposure. Studies exploring the relationship between exposure to power EMF from HVT lines and neurobehavioral function in children are insufficient. Studies in adults and animal models suggest that acute cognitive effects may occur from short-term exposure to intense EMF. Characterization of these effects is necessary for the development of exposure guidelines. Unfortunately, there is a lack of studies investigating field-dependent effects in children [15].

In children, exposure to environmental contaminants varies and, in many cases, is much higher than adults [16]–[18]. Exposure differences in children are due, in part, to differences in physiological function, surface-to-volume ratio and behavior [19]. Children are more sensitive to EMF compared to adults because they are still in the physiological and psychological development period. The nervous system has bioelectric properties that make it more susceptible to the effects of EMF [20]. Generally, children in developing countries experience more intensive EMF effects than children of more developed countries due to greater clustering of homes around very high voltage lines [21]. The biophysical mechanism of acute exposure to high levels EMF is clear. Currently, intense debate is focused on whether long-term, low-level EMF exposure below exposure limits can cause adverse health effects or influence well-being. Gaining a greater understanding of the effects of EMF on children, especially impact on early development, remains to be a primary challenge [22]. The present study aims to explore the influence of EMF from 500 kV HVT lines on neurobehavioral function in children.

## Materials and Methods

### Ethics Statement

This study was approved by the Ethical Review Committee for Environmental Health and Related Product Safety, Chinese Center for Disease Control and Prevention (No. 201203) and written informed consent was obtained from the parents of all children who participated in the survey. We conducted field studies at or around School A and School B. No specific permission was required for any locations/activities involved in this study. Because none of School A and School B is located in a national park or other protected area of land, the relevant regulatory body concerned with protection of wildlife, private land, etc. and the field studies did not involve endangered or protected species. School A and School B were located in Guangzhou of Guangdong Province of China. The two schools belong to public schools. Our study was approved by the local education departments and schoolmasters of the two participating schools.

### Selection of Study Areas

According to the national principles of students automatically going to neighborhood schools, in Guangzhou city, children attended a primary school by their geographic district. This could guarantee the students in the same school lived in similar living environment. In our study, the average distance between children’s households and their school were within 2 km, and majority of them lived within 4 km from their schools. Resident areas of selected students in school A were located approximately 12 km away from the areas of students selected in school B. Because of being belong to the same town and the close distance from each other, the residents in different villages have similar living environment and eating habits.

Before our formal investigation, we had collected data of various environmental factors, including air pollution, noise and green area around each school. Both of the two primary schools were located in the suburb of Guangzhou, where few arterial roads located and there are very few traffic jams. According to the daily air quality reports on website of Guangzhou environmental monitoring station, the air quality in the areas is good. There are no local industrial pollution sources around each school. Additionally noise and green area are similar in the two schools.

Study areas were selected based on the monitoring data of ambient power frequency EMF intensity for Guangzhou, Guangdong Province, China, and local HVT lines distribution. According to our careful field visit and environmental EMF intensity monitoring data, we selected our study areas. School A had no HVT lines in close proximity within 4 km. School B, located in an area, was 94 m away from 500 kV HVT lines. Otherwise, there were no other EMF sources, such as TV tower, mobile phone base stations around the two schools. According to the monitoring data of ambient power frequency EMF intensity for each school, it showed that the intensity of power frequency EMF in School A was close to environmental background levels. However, nearly half of all measurements at School B were in the range of 0.2–0.4 µT. Intensity of power frequency EMF in School B was significantly higher than that in School A. In order to define explicitly different levels of exposure to EMF from HVT lines of the two schools in our study, School B was defined to locate in a higher power frequency EMF exposure area compared with school A.

### Monitoring Intensity of Ambient Power Frequency EMF

Intensity of power frequency EMF was measured at each school according to environmental protection industry standards of the People’s Republic of China, which named the Guidelines on Management of Radioactive Environmental Protection Electromagnetic Radiation Monitoring Instruments and Methods (HJ/T10.2-96) [23] and the Technical Regulations on Environmental Impact Assessment of Electromagnetic Radiation Produced by 500 kV Ultrahigh Voltage Transmission and Transfer Power Engineering (HJ/T24-1998) [24]. Intensity of ambient power frequency EMF was monitored by an EFA-300 power frequency electromagnetic field strength tester (Narda in German). Measuring sites included classrooms and the main activity areas of students, such as corridors outside classrooms, a basketball court and a playground. Spot measurements were acquired only on days where temperatures ranged from −10–40°C and humidity values ranged from of 0–80%. According to the average height of students, the measuring probe was consistently placed at a height of 1.2 m from the ground. Both electric field intensity (V/m) and magnetic field intensity (nT) of 50 Hz were measured. Each measurement was acquired over a minimum period of 15 s. Upon stabilization of a reading, the maximum value was recorded. Continuous measurements were performed in triplicate at each measuring site, and the average was reported. Additional sampling details, such as measurement time, location, direction and weather conditions, were also recorded.

### Identification and Selection of Participants

School A and School B belong to a same town and both of them are public elementary schools which have comparable teaching level according to information from local education authority. There are three or four classes in each grade in both School A and School B. There are about forty students in each class. Field work was conducted on November 15–16, 2011. Children from fourth to sixth grade (9–13 years of age) in each school (n = 437; 225 from School A and 212 from School B) were selected to participate in a questionnaire survey and computerized neurobehavioral testing. In School A, 225 students came from three fourth grade classes, two fifth grade classes and one sixth grade class. In School B, 212 students came from same grade classes as School A.

Written informed consent was obtained from the parents of all children who participated in the survey. Eligibility criteria for selecting student participants were as follows: a) length of residence in the area for 2 or more years, b) no reported psychoses or known neurologic hereditary diseases, c) good health at time of testing, d) a completed questionnaire, and e) parental or legal guardian permission to participate in the study. Before we conducted computerized neurobehavioral tests, exclusion criteria was in strict accordance with the WHO’s recommendation about the Neurobehavioral Core Test Battery (NCTB). Exclusion criteria for selecting student participants were as follows: a) diseases in nervous-mental system, b) students can not complete tests independently due to vision disorder, hearing disorder, or hand movement disorders, c) Alcohol consumption, taking analeptic or sedatives 4 hours before tests. Additionally, the medical examination of students was conducted by qualified physicians.

### Questionnaire Survey

Self-reporting questionnaires were distributed to students by head teachers and filled out by their parents or legal guardians. An introductory letter was included with the questionnaires, informing parents of the main goals and activities of the project and noting that they had the right to decline participation of their child if they so desired. Parents were also informed that they did not have to answer all questions in the questionnaire.

The questionnaire consisted of two sections. Part 1 acquired socio-demographic and socioeconomic data of the children, such as age, sex, years of education, residential history, household income, tobacco and alcohol consumption, and living conditions. Part 2 surveyed student exposure to EMF from sources other than HVT lines at schools, such as household appliances (computers, TVs, mobile phones and fixed-line telephone) or high-tension lines and transformer substations near the residence, experienced by students.

In the Part 2 of the questionnaires, perceived proximity to power lines and transformer/transformer substation were investigated with question as follows: “Are there any high-tension lines surrounding your residence” and “Are there any transformer/transformer substation surrounding your residence” (“surrounding” was defined as perceived distance from residence to power lines/transformer/transformer substation within 500 m), respectively. Answers were categorized as “Yes” and “No” reflecting a high and low perception of proximity respectively. Four different household appliances, namely, computers, TVs, mobile phones and fixed-line telephone were selected a priori as the most frequent use by pupils. About the use of these household appliances starting with a question as follows: “Is use of …frequent in your daily life? ” The use of each appliance was recorded on two levels, namely “Yes” and “No” as a result. “Frequent” was defined according to different characteristic of each appliance. It was defined as “more than two hours a day on average” for computers, TVs and mobile phones, while “more than half an hour a day on average” for fixed-line telephone. Additionally, a question, namely “Do your bed located near air conditioner or refrigerator” was also in the investigation. “Near” was defined as “Distance from bed to air conditioner/refrigerator is within 2 m.”

### Computerized Neurobehavioral Testing

With the understanding that children differ from adults in their comprehension and cognitive ability, only four computerized tests were chosen from the Computerized Neurobehavioral Evaluation System (4th Chinese version; NES-C4). All tests were conducted in a classroom under the guidance of trained investigators. All investigators were blinded to children exposure status. Standard oral instructions were provided to ensure that all children fully understood the testing process. Furthermore, no children were given the real test until passing the simulated pretest. Throughout testing, children were instructed to answer questions and ask for assistance if necessary. Four tests were conducted as follows:

#### Visual Retention Test (VRT)

A geometric figure was displayed on the computer screen and children were instructed to memorize the shape and other defining characteristics. The figure was then replaced by a display of four figures (labeled 1 through 4), only one of which was the original geometric figure. As quickly as possible, children were instructed to press the number key corresponding to the original figure. A total of 15 trials were administered with a duration of 4 s each.

#### Visual Simple Reaction Time (VSRT)

A red square was displayed 20 times on a computer screen for a duration of 2 s each time. Children were asked to press the spacebar on the keyboard with their preferred hand as soon as they saw the red square on the screen. The trial was then repeated with the non-preferred hand.

#### Digit Symbol (DSB)

Children were given a sheet of paper with a grid that labeled nine different symbols from 1 to 9. An empty grid with only the numbers (1 through 9) was presented on another sheet, and the children were instructed to write down the matching symbol for each number as quickly as possible. A trial time of 90 s was provided. Each correct answer resulted in a score of 1, and a total score consisting of the sum of correct answers was recorded.

#### Pursuit Aiming Test (PAT)

The children were presented with a piece of paper with small circles on it and instructed to place a dot in the center of each circle as quickly as possible. The number of dots correctly placed was recorded.

### Statistical Analysis

For computerized neurobehavioral testing, scores were expressed as a Neurobehavioral Ability Index (NAI), which is an integrated index directly computed by NES with the following formula [25]:
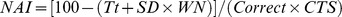
where *Tt* is the total time (in seconds), *SD* is the standard deviation of the correction coefficient (0.116 sec/time), *WN* is the number of wrong performance times, *Correct* is the number of correct performance times, and *CTS* is the *SD* of average time of correct performance (sec/time).

All data was entered into a single computer file, and all statistical analyses were conducted using SPSS version 16.0 software (SPSS Inc., Chicago, IL, USA). Spot measurements data was skewed distribution, therefore, Two-independent Samples Nonparametric Test was used in order to compare different EMF exposure levels between School A and School B. Socio-demographic characteristics and socioeconomic factors of participants included both continuous variables and categorical variables. Variables investigated in the questionnaire survey of EMF exposure from sources other than HVT lines were all categorical variables. One-way analysis of variance (ANOVAS) was used for continuous variables, while a chi-square test or Fisher’s exact test was used to compare categorical variables. Associations between power frequency EMF exposure and NAI scores were analyzed using One-way ANOVAS and multinomial linear regression model. 4 different ANOVAS and 4 multiple linear regression models were done considering NAI scores of VRT, DSB, VSRT and PAT as dependent variables. Backward selection for independent variables with the entry significance level set to 0.05 and a removal level of 0.10 was used in the multinomial linear regression model. Eleven independent variables were included in the regression model. The independent variables included in multiple linear regression models were selected based on previous studies on neurobehavioral functions [26]–[31] and univariate analysis from the current study. These independent variables included age(years), body mass index(BMI), years of education(years), length of residency(years), exposure status(0 = school A, 1 = school B), sex(0 = male, 1 = female), second-hand smoking (0 = no,1 = yes), familiarity with computer games (0 = little to none, 1 = much), sleep status(0 = bad, 1 = not bad, 2 = good), household fuel (0 = coal, 1 = liquified petroleum gas, 2 = electricity, 3 = others), high-tension lines surrounding residence (0 = no,1 = yes) and frequent use of household appliances (0 = no,1 = yes).

## Results

### Intensity of Electric and Magnetic Fields

Monitoring data from the two schools included the study showed that intensity of electric fields at both School A and School B were far below the maximum exposure limit (4000 V/m) for public electric fields. Median electric field intensity at School A and School B were 0.417 V/m (ranging from 0.016 V/m to 2.919 V/m) and 1.34 V/m (ranging from 0.522 V/m to 3.93 V/m), respectively. A comparison of electric field intensity between the two schools was statistically significant (Z = −3.981, p<0.001) (Table 1).

**Table 1:** Power frequency electric field measurements (V/m).

School	N	M±IR	Min	P_25_	P_50_	P_75_	Max	Reference limit#
School A	30	0.417±0.84	0.016	0.108	0.417	0.949	2.919	4000 V/m
School B	21	1.34±2.83**	0.522	0.627	1.340	3.46	3.930	4000 V/m

# Reference limit came from the Technical Regulations on Environmental Impact Assessment of Electromagnetic Radiation Produced by 500 kV Ultrahigh Voltage Transmission and Transfer Power Engineering (HJ/T24-1998) in China.

** Two-Independent Samples Nonparametric Test, Z = 3.981, p<0.001.

N = number of spot measurements; M = median = P_50_; IR = interquartile range = P_75_–P_25_.

Though all measurements obtained for magnetic fields were lower than reference limit, the intensity of power frequency magnetic fields at School B was significantly higher than School A (*Z* = −6.029, p*<*0.001). Median intensity of magnetic fields at School A and School B were 0.028 µT and 0.20 µT, respectively. The highest measurements obtained at School A and B was 0.072 µT and 0.36 µT, respectively. Nearly half of all measurements at School B were in the range of 0.2–0.4 µT (Table 2).

**Table 2:** Power frequency magnetic field measurements (µT).

School	N	M±IR	Min	P_25_	P_50_	P_75_	Max	Reference limit#
School A	30	0.028±0.027	0.010	0.016	0.028	0.043	0.072	0.4 µT
School B	21	0.20±0.11**	0.17	0.19	0.20	0.31	0.36	0.4 µT

# Reference limit came from “Assessment conclusions and suggestions of WHO’s international EMF project”.

** Two-Independent Samples Nonparametric Test, Z = 3.981, p<0.001.

N = number of spot measurements; M = median = P_50_; IR = interquartile range = P_75_–P_25_.

### Socio-demographic Characteristics of Participants

A questionnaire was used to survey 437 students at two primary schools. Among the 437 students, 10 students were excluded from the survey for the following reasons: length of residence less than 2 years (6 students; 3 each from School A and B), invalid questionnaire responses (3 students from School A), or a history of epilepsy (1 child from School A). The remaining 427 students (206 from School A and 221 from School B), who completed the questionnaire and computerized testing, met study inclusion criteria. We presented the characteristic of the participant in two different tables (Table 3 and Table 4). Table 3 presents means (Mean ± SD) and Table 4 presents frequencies [n (%)].

**Table 3:** Socio-demographic characteristics of participants (Mean ± SD).

Characteristics	Students in School A	Students in School B	*F* ^a)^	p-value
Age, years	11.33±1.35	10.63±0.98	38.576	<0.001
Height, cm	146.05±10.09	141.57±8.12	25.327	<0.001
Weight, kg	37.95±8.77	34.84±9.07	12.884	<0.001
BMI, kg/m^2^	17.60±2.61	17.17±3.05	2.397	0.122
School age, years	6.31±0.64	6.35±0.61	0.587	0.444
Years of education, years	8.61±1.51	7.77±1.06	44.399	<0.001
Length of residency, years	10.86±2.29	10.11±2.21	12.099	<0.001

^a)^One-way analysis of variance was used for continuous variables; BMI = body mass index.

**Table 4:** Socio-demographic characteristics of participants [n (%)].

Characteristics	Students inSchool A		Students in School B		Test statistic e)	p-value
	n a)	% b)	n c)	%^ d)^		
Sex						
Male	115	55.83	103	46.61	*χ^2^* = 3.626	0.057
Female	91	44.17	118	53.39		
Nationality						
Han nationality	204	99.03	217	98.20	*Fisher’s exact test*	0.686
Minority	2	0.97	4	1.80		
Household income(RMB¥)^ f)^						
≤3,000	56	27.18	59	26.70	*χ^2^* = 0.584	0.900
3,001–10,000	46	22.33	51	23.08		
10,001–50,000	95	46.12	98	44.34		
>50,000	9	4.37	13	5.88		
Smoking						
Yes	0	0	0	0	*–*	–
No	206	100.0	221	100		
Drinking						
Yes	0	0	3	1.36	*Fisher’s exact test*	0.249
No	206	100.0	218	98.64		
Second-hand smoke						
Yes	116	56.86	133	60.45	*χ^2^* = 0563	0.453
No	88	43.14	87	39.55		
Familiarity with computer games						
Little to none	186	90.30	200	90.50	*χ^2^* = 0.005	0.942
Much	20	9.70	21	9.50		
Sleep status						
Bad	41	19.90	57	25.79	*χ^2^* = 2.767	0.251
Not bad	34	16.51	40	18.10		
Good	131	63.59	124	56.11		
Household fuel						
Coal	24	11.94	16	7.41	*χ^2^* = 3.809	0.283
Liquefied petroleum gas	34	16.92	48	22.22		
Electricity	110	54.73	119	55.09		
Others	33	16.41	33	15.28		

a) Dada may not sum up to n = 206 in School A due to non-response.

c) Dada may not sum up to n = 221 in School B due to non-response.

b) and^ d)^ Dada sum up to 100% due to calculation not included non-response.

e) A chi-square test or Fisher’s exact test was used to compare categorical variables.

^f)^Monthly income per capita.

Students from School A and School B had a similar distribution for BMI, school age, sex, nationality, household income, familiarity with computer games, sleep status, exposure to second-hand smoking and type of household fuel. Although students from School B had significantly lower height and body weight than those from School A, BMI showed no significant difference. However, children from School A were older and had more years of education and length of residency compared with those from School B. A majority of participants in both groups reported that their families used electricity for cooking (54.73% in School A and 55.09% in School B). Although the average length of residency for students in School A (10.86±2.29 years) was statistically different from students in School B (10.11±2.21 years), all students involved in the current study had lived in their current household for more than 2 years. The overwhelming majority of students were local residents who lived in their current household since birth. Specifically, 95.6% and 93.7% of students from School A and School B, respectively, had lived in their current household for more than 5 years. In addition, children from School A reported 8.61±1.51 years of education, whereas those from School B reported 7.77±1.06 years (p<0.001; Table 3).

### Investigation of EMF Exposure from Sources other than HVT Lines

Table 5 compares students’ exposure to EMF from daily activities outside of school. Both groups reported similar exposure from “high-tension lines surrounding residence”, “transformer or transformer substations surrounding the residence”, “transformer or transformer substation from residence to school ”,“bed located near air conditioner or refrigerator” as well as the average use of household appliances, computers and mobile phones. A higher proportion of students from School B reported watching more TV (p = 0.031), but less frequent use of fixed-line telephones (p = 0.007).

**Table 5:** Investigation of EMF exposure from sources other than HVT lines.

sources	Students in School A		Students in School B		*χ^2^*	p-value
	n a)	% b)	n c)	%^ d)^		
High-tension lines surrounding residence						
Yes	98	47.57	114	52.05	0.853	0.356
No	108	52.43	105	47.95		
Transformer or transformer substation surrounding residence						
Yes	49	23.90	52	23.85	<0.001	0.991
No	156	76.10	166	76.15		
Transformer or transformer substation from residence to school						
Yes	60	29.56	64	28.96	0.018	0.893
No	143	70.44	157	71.04		
Frequent use of household appliances e)						
Yes	85	42.08	90	42.65	0.014	0.906
No	117	57.92	121	57.35		
Frequent use of computers						
Yes	88	43.14	116	52.49	3.717	0.054
No	116	56.86	105	47.51		
Frequent use of TVs						
Yes	147	72.41	135	62.50	4.674	0.031*
No	56	27.59	81	37.50		
Frequent use of mobile phones						
Yes	47	23.50	44	20.47	0.557	0.455
No	153	76.50	171	79.53		
Frequent use of fixed-line telephone						
Yes	52	25.87	32	15.09	7.395	0.007*
No	149	74.13	180	84.91		
Bed located near air conditioner or refrigerator						
Yes	27	13.30	36	16.59	0.890	0.345
No	176	96.70	181	83.41		

a) Dada may not sum up to n = 206 in School A due to non-response.

c) Dada may not sum up to n = 221 in School B due to non-response.

b) and^ d)^ Dada sum up to 100% due to calculation not included non-response.

e) It was defined as the use of one or more of the household appliances, namely computers, TVs, mobile phones and fixed-line telephone in daily life.

* Compared with students in School A, P<0.05.

### Computerized Neurobehavioral Testing

Children from School A had higher NAI scores in all items tested except for VRT. Analysis of variance (ANOVA) showed a significant differences for VSRT NAI scores (p<0.05) in the Table 6. Table 7 summarizes the results from multiple linear regression models. After controlling for potential confounding factors, exposure status, age, sex, years of education, familiarity with computer games and sleep status were found to be significantly associated with students’ NAI. The model indicated that older students received higher VSRT and PAT NAI scores than younger students. Moreover, boys tended to obtained higher VRT and PAT NAI scores than girls. In general, more years of education were a key factor for obtaining higher VRT, PAT and DSB NAI scores. Greater familiarity with computer games was also significantly associated with higher VRT and PAT NAI scores, and better sleep was significantly associated with higher DSB NAI scores. More interestingly, results showed that students from School B (a higher power frequency EMF exposure area) had lower VRT (p = 0.002) and PAT (p<0.001) NAI scores, suggesting that exposure to EMF from HVT lines might have a significant influence on neurobehavioral function.

**Table 6:** ANOVA results from computerized neurobehavioral testing.

Test a)	Students in School A		Students in School B		*F*-test	p-value
	No.	Mean ± SD	No.	Mean ± SD		
VRT	206	1.04±0.44	221	1.06±0.42	0.105	0.746
DSB	203	2.67±1.87	214	2.32±1.84	3.704	0.055
VSRT	205	1.41±0.13	221	1.37±0.17	5.937	0.015*
PAT	205	1.82±0.65	221	1.81±0.52	0.068	0.795

a) 4 different ANOVAS were done considering NAI scores of VRT, DSB, VSRT and PAT as dependent variables.

* Compared with students in School A, P<0.05.

VRT = visual retention test; DSB = digit symbol; VSRT = visual simple reaction time; PAT = pursuit aiming test.

**Table 7:** Summary of results from multiple linear regression models.

Independent variable a)	VRT b)		DSB b)		VSRT b)		PAT b)	
	Beta	p-value	Beta	p-value	Beta	p-value	Beta	p-value
Exposure status	−0.161	<0.001*	−0.018	0.727	−0.048	0.346	−0.186	<0.001*
Age	0.100	0.101	−0.045	0.504	0.268	<0.001*	0.146	0.011#
Years of education	0.349	<0.001*	0.236	<0.001*	0.077	0.245	0.406	<0.001*
Familiarity with computer games	0.179	<0.001*	0.063	0.201	0.057	0.240	0.24	<0.001*
Sex	−0.194	<0.001*	−0.007	0.886	−0.028	0.566	−1.05	0.012#
Length of residency	−0.002	0.973	−0.075	0.155	−0.020	0.716	−0.003	0.948
BMI	−0.053	0.251	0.035	0.485	0.071	0.151	0.005	0.913
Second-hand smoke	0.025	0.582	−0.031	0.523	−0.026	0.593	−0.021	0.618
Sleep status	0.014	0.751	0.110	0.016#	0.033	0.496	−0.020	0.636
Household fuel	0.034	0.457	0.023	0.644	0.021	0.666	−0.063	0.134
High-tension lines surrounding residence	−0.097	0.062	0.029	0.553	0.010	0.841	−0.068	0.105
Frequent use of household appliances	−0.016	0.719	−0.030	0.545	0.001	0.986	0.024	0.573

a) Independent variables included exposure status(0 = school A, 1 = school B), age(years), years of education(years), familiarity with computer games (0 = little to none, 1 = much), sex(0 = male, 1 = female), length of residency(years), body mass index(BMI), second-hand smoking (0 = no,1 = yes), sleep status(0 = bad, 1 = not bad, 2 = good), household fuel (0 = coal, 1 = liquified petroleum gas, 2 = electricity, 3 = others), high-tension lines surrounding residence (0 = no,1 = yes) and frequent use of household appliances(0 = no,1 = yes).

b) 4 different multiple linear regression models were done considering NAI scores of VRT, DSB, VSRT and PAT as dependent variables. Backward selection for independent variable was used in the model with the entry significance level set to 0.05 and a removal level of 0.10.

# Independent variable reserved in the model was with a p-value <0.05;

* Independent variable reserved in the model was with a p-value <0.001.

Beta = standardized coefficients; VRT = visual retention test; DSB = digit symbol; VSRT = visual simple reaction time; PAT = pursuit aiming test.

## Discussion

### Neurobehavioral Function in Children

To date, no epidemiological research has been published on the contribution of EMF from HVT lines to neurobehavioral function in children. Epidemiological studies on the effects of power EMF on neurobehavioral function have primarily focused on occupational exposure. No study has been conducted to investigate the issue in children in a developing country. The present study found children in a school located in a higher power frequency EMF exposure area (School B) had lower NAI scores than those from a school located far away from HVT lines (School A). After adjusting for potential confounding factors, namely age, sex, BMI, years of education, familiarity with computer games, length of residency, second-hand smoke, sleep status, household fuel, high-tension lines surrounding residence and frequent use of household appliances, significant associations between power EMF exposure and poor performance on neurobehavioral VRT and PAT tests were found, indicating lower psychomotor, motor, and sensory function.

A study conducted by Qin et al. [32] reported neurobehavioral function of electricians working under power frequency electric fields may be impaired to a certain extent. Long-term exposure to power frequency electric fields may induce neural symptoms. Conversely, Nevelsteen et al. [33] reported that an acute exposure to ELF magnetic fields does not affect cognitive performance parameters, such as mood, vigilance, and reporting of symptoms. Two double-blind studies by Crasson et al. [34] indicated that low-level 50 Hz magnetic fields may have a slight influence on event-related potentials and reaction time under specific circumstances of sustained attention. Another double-blind study in healthy young men conducted by Delhez et al. [35] reported that no effect was observed after ELF magnetic fields exposure on cognitive tests. Kurokawa et al. [36] examined acute effects of 50 Hz magnetic fields on cognitive performance in humans, and found no association. After reviewing possible effects of low frequency fields on human cognition and performance, the ICNIRP report [37] concluded that some changes of magnetic fields on reaction time and accuracy have been reported but the effects were not consistent between studies and further studies are required to clarify possible effects on reaction time and accuracy. These conclusions were similar to a review conducted by Crasson [38], in which the authors thought that we cannot exclude the possibility of 50–60 Hz weak magnetic field exposure on human cognitive processes.

The mechanism of interaction between ELF-EMF and biological systems, especially the nervous system, are areas of intense research focus [39]–[45] and it has been controversially discussed. Exposure to ELF-EMF can induce electric fields and currents within the body, but almost always much lower than those that stimulate peripheral nerve tissue. The integrative properties of synapses and neural networks of the central nervous system render cognitive function sensitive to the effects of physiologically weak EMF below the threshold for peripheral nerve stimulation. Thermal and non-thermal interaction mechanisms may be the bioeffects about weak EMF, which are characterized by a threshold field strength (below which no observable response is produced) and dynamics. So far, the health impacts of low-level ELF-EMF on humans cannot be explained clearly. Further research is needed to confirm reports of the effects of weak fields and determine the relevance of these effects to human health [46], [47].

### Validity of Computerized Neurobehavioral Tests in Children

The Neurobehavioral Core Test Battery (NCTB) recommended by the WHO in 1986 consists of seven tests, which test the emotional state, short-term memory, coordination of movement and reaction rate of behavior functions in subjects. NCTB aimed to unify the evaluation method of early nerve damage in population of occupational exposure. Because the NCTB is standardized and easy to administer, it has been widely used in the field of preventive medicine.

Computerized Neurobehavioral Evaluation System (NES) was built on the basis of NCTB. It was designed for occupational studies in adult workers, but many NES tests can be completed by children as young as 7 or 8 years of age. Some NES tests, such as simple reaction time, can even be completed by preschool children. Many countries, including the United States, Spain and the Philippines, have investigated the feasibility and reliability of NES testing in children [26]–[31]. These studies concluded that NES can be used to evaluate neurobehavioral function in children, but age, sex, years of education and computer proficiency influence test results. In general, older children receive higher scores, and boys score better than girls. Furthermore, children familiar with computers perform better than those without such experience [30], [31]. As such, study investigators selected appropriate NES tests based on different psychological and physiological characteristics between children and adults. Control for confounding factors, such as age, sex and familiarity with computers, was also required [48]. In the end, four NES tests were administered to children in the current study: Visual Retention Test (VRT), Digit Symbol (DSB), Visual Simple Reaction Time (VSRT) and Pursuit Aiming Test (PAT).

### Exposure of Children to Power Frequency EMF from HVT Lines at School

An EMF is composed of two components, an electric and magnetic field. Electric and magnetic fields have different properties that are of importance when considering possible biological effects. Essentially all materials, including clothing, can easily shield power frequency electric fields. In contrast, the properties of magnetic fields can pass through almost all materials, including living tissues, building structures and earth [22]. Thus magnetic fields are more closely linked to human health than electric fields. A report by the WHO suggests that intensity of power frequency electric fields from power lines are close to environmental background values from a distance of 50 m. The same report also suggests that residential power frequency magnetic fields are associated with the proximity of high voltage power lines, advocating that residences, schools, nursery schools, and hospitals not be located within 50 m of high voltage power lines [49]. Intensity of power frequency magnetic fields from HVT lines is the greatest directly under the line. When the distance from high tension lines is greater than 100 m, the intensity of magnetic fields is close to environmental background values [50]. In the current study, the intensity of power frequency electric fields in each primary school was far below the recommended maximum exposure limit, possibly due to the great distance between the schools and HVT lines or simply that all electric fields emitted were easily shielded.

Pooled analyses of epidemiological studies demonstrated a consistent two-fold increase in childhood leukemia associated with average exposure to residential power frequency magnetic fields above 0.3–0.4 µT [4], [51]. Current recommendations to limit effects on nervous system function for 50/60 Hz electric fields are 2 mA/m^2^ current density; for 50 Hz power frequency fields, this translates to 5 kv/m for electric fields and 100 µT magnetic fields [51]. The EMF guidelines set by International Commission on Non-Ionizing Radiation Protection (ICNIRP) are based on shock hazards, not cancer or other health effects. Several government authorities, such as the Swedish Board for Safety, have issued statements proposing a reduction in general exposure to EMF and placement of schools and daycare centers only in locations where magnetic fields do not exceed 0.2–0.3 µT [52]. Uncertainties regarding a potential causal association between exposure to ELF-EMF fields and adverse health outcomes have led some to suggest that prudent avoidance of EMF exposure may be justified [52]–[54]. For this reason, study investigators chose to use 0.4 µT as the reference limit for magnetic field exposure.

Children, who spend a majority of their time at school facilities, are more sensitive to EMF than adults. In the present study, the distance of School B from 500 kV power lines was 94 m. Compared to the reference limit (0.4 µT), the overall intensity of power frequency magnetic fields at the two schools was lower, but the median measurement at School B was 0.2 µT, while the maximum value recorded was close to 0.4 µT. Studies [55], [56] found that children’s exposure to magnetic fields at a school located close to the power lines influenced considerably the time-weighted average exposure to magnetic over 24 h. Furthermore, the power line was the most important source of exposure when the magnetic field was greater than about 0.2 µT [55]. Therefore, we can not afford to ignore the students’ EMF exposure in the School B. Health effects related to short-term, high-level exposure are well-documented and have led to the establishment of two international exposure limit guidelines [51], [57]. At present, possible health effects due to long-term, low-level exposure to ELF-EMF have insufficient scientific evidence to justify lowering quantitative exposure limits.

### Exposure to Magnetic Fields from other Sources Outside School Environment

Time pattern of pupils whose time mostly consists of that at school and that at home is much simpler and more relatively fixed that the time pattern of adults. Exposure to magnetic fields from other sources outside school environment, especially home environment, may have contribution to the total exposure. Sources of residential power-frequency magnetic fields mainly included five sources, namely HVT lines, electric power distribution lines, ground currents, home wiring, and household appliances, in which HVT lines and home appliances played an important role [58].

In our questionnaire, we investigated perceived proximity to power lines around children’s residences. Results showed that the distance from children’s residences to transmission lines within 500 m seems to concern a relatively high fraction of the selected children both in School A and School B (47.57% and 52.05%, respectively). Relatively little quantitative information on children’s residential exposure to magnetic fields from HTV lines is available in the study. However, the number of buildings very close to the power lines is low [59], [60] and increase constantly up to a distance of about 100 m [59]. Household appliances might be the main source of residential exposure to 50 Hz magnetic fields in children’s home environment in our study.

The exposure assessment and contribution of home appliances to residential exposure to magnetic fields has been questioned for years [61]–[63]. Questionnaires have been used to assess exposure to the EMF produced by home appliances. The results of questionnaires about children’s house appliances showed that TVs watching and the use of fixed-line telephone were statistically significant between school A and school B. But the EMF generated from TVs and fixed-line telephone was weak and mostly less than 0.1 µT [62].

Although exposure to magnetic fields of household appliances are not negligible in daily life [63], only some appliances, such as electric blankets, hair dryers, curling irons, and electric razors can deliver substantial short-term partial-body exposures to their users [62], [64]. The fields produced by most household appliances were all less than 0.1 µT at distances from them exceeding 1 m [65]. However, these household appliances were not be used popularly in children attending a primary school. For a pupil, household appliances, such as computers, TVs, mobile phones and fixed-line telephone are frequent used. Although one child had greater exposure in some specific situations, such as playing computer games, cooking, and, approaching the television to switch the set on or off [55], [66], exposure was rather stable at home and that the children were only seldom exposed to magnetic filed from household appliances due to pattern of use of a child and generated relatively small fields [55], [62].

### Study Limitations

Firstly, only students from fourth to sixth grade were selected to participate in the study due to some limitations during the fieldwork. Though 93.8% of children selected from the two schools were allowed to participate in our study, we could not collect more information about the pupils who did not like to participate because of no questionnaires returned from them. So we could not compare information between participants and nonparticipants. Moreover, the presence of a potential source in EMF in a school surrounding might have an effect on participation rate of students. This potential selection bias might distort the true association between the EMF exposure from HVT lines and the outcomes of neurobehavioral function in children.

Secondly, only spot measurements were taken to assess exposure of children at school and questionnaires were used to assess exposure to the EMF outside the school environment in the present study. Methods for exposure assessment remain among the most influential determinants of study quality [67]. The study [68] conducted by Frei P et al. showed that personal exposure correlated best with the full exposure prediction model and spot measurements. Questionnaire-based information on appliance use has limited value in the personal exposure to magnetic fields [62]. We had try to control for the home environment between students in school A and those in school B. Actual distance from EMF sources to home of the participant, as the housing parameters should be taken into account. However, the 437 students selected lived at different homes. We did not monitor the actual power frequency EMF intensity at each home for them due to limitations of time and funds. Therefore, quantification of exposure from household appliances was missing in our study. The shortcomings of exposure assessment in the study might results in some uncertainty.

Furthermore, since the cognition and comprehension ability of children differs significantly from adults, it is unclear if student participants fully understood the meaning of every question in the self-reported questionnaire survey. This shortcoming may lead to bias in results. Additionally, this cross-sectional epidemiological study selected only two exposure groups for study inclusion. The sample size might be relatively small. And the covariates that were considered in the study were limited, many other residual contributing factors may have been ignored. Therefore, the outcomes in the present study might not be generalizable to all children.

Finally, time-activity patterns could potentially confound associations between EMF exposure and the children’s health outcomes, but we did not have sufficient data to address this topic. As potential limitations mentioned above in the study, inferences of causality cannot be made, and further studies are required to confirm the results.

### Conclusions

The results of the current study suggest that there is a significant association between power EMF exposure and poor performance on neurobehavioral VRT and PAT tests, indicating that long-term low-level exposure to power EMF from HVT lines may have a negative impact on neurobehavioral function in children. However, prudence is suggested in generalizing these results to the other children due to the reasons as follows: a) Results from the multivariate analysis suggested that exposure status was only significantly different with respect to half of all the four tests. Association between power EMF exposure from HVT lines and poor performance on neurobehavioral was not strong. b) In addition, potential limitations, such as selection bias, lack of exposure assessment and relatively small sample size will limit generalization of these results.

Although it is unclear if these results imply a risk, it appears that such EMF exposures at School B are unusually high for school environment. Although few pupils attend schools close to such a HVT line and the possible attributable health risk from this exposure in the general children is probably low, but the relative risk for the few exposed children might be important. Our results indicate further research is needed to clarify the potential risks of ELF-EMF on neurobehavioral function and development in children.
